# Coronavirus-positive Nasopharyngeal Aspirate as Predictor for Severe Acute Respiratory Syndrome Mortality

**DOI:** 10.3201/eid0911.030400

**Published:** 2003-11

**Authors:** Owen Tak-Yin Tsang, Tai-Nin Chau, Kin-Wing Choi, Eugene Yuk-Keung Tso, Wilina Lim, Ming-Chi Chiu, Wing-Lok Tong, Po-Oi Lee, Bosco Hoi Shiu Lam, Tak-Keung Ng, Jak-Yiu Lai, Wai-Cho Yu, Sik-To Lai

**Affiliations:** * Princess Margaret Hospital, Hong Kong; † Public Health Laboratory Centre, Hong Kong

**Keywords:** Severe acute respiratory syndrome, SARS virus, coronavirus infection

## Abstract

Severe acute respiratory syndrome (SARS) has caused a major epidemic worldwide. A novel coronavirus is deemed to be the causative agent. Early diagnosis can be made with reverse transcriptase-polymerase chain reaction (RT-PCR) of nasopharyngeal aspirate samples. We compared symptoms of 156 SARS-positive and 62 SARS-negative patients in Hong Kong; SARS was confirmed by RT-PCR. The RT-PCR–positive patients had significantly more shortness of breath, a lower lymphocyte count, and a lower lactate dehydrogenase level; they were also more likely to have bilateral and multifocal chest radiograph involvement, to be admitted to intensive care, to need mechanical ventilation, and to have higher mortality rates. By multivariate analysis, positive RT-PCR on nasopharyngeal aspirate samples was an independent predictor of death within 30 days.

Severe acute respiratory syndrome (SARS) is an emerging infectious disease worldwide. By May 28, 2003, a total of 745 patients had died of SARS and 8,240 persons were infected. At the same time**,** 270 patients had died of SARS and 1,730 were diagnosed in Hong Kong. As defined by the Centers for Disease Control and Prevention (CDC), a suspected SARS patient is a person with a temperature >38°C; clinical findings such as cough, shortness of breath, and difficulty breathing, together with history of travel to an area with documented local transmission or close contact with a suspected SARS patient within 10 days of symptoms onset. A probable SARS case also requires radiologic evidence of pneumonia or respiratory distress syndrome or autopsy findings consistent with pneumonia or respiratory distress syndrome without an identifiable cause (*1*). Because of this nonspecific definition, many non-SARS patients may be mislabeled as having SARS. The discovery of coronavirus as the causative agent and the establishment of laboratory tests for coronavirus have aided the research direction. However, these tests only act as supplementary aids to the diagnosis of suspected and probable cases of SARS. The diagnostic tools for coronavirus infection include reverse transcriptase-polymerase chain reaction (RT-PCR), serologic testing, electron microscopy, and viral culture. A fourfold increase in paired serologic test results suggest highest sensitivity and is regarded as the standard criterion for diagnosis. Studies have shown that antibodies against coronavirus are usually present 14–21 days after onset of symptoms. Electron microscopy and viral culture for coronavirus are specific, but the sensitivity is low. The sensitivity of the RT-PCR for coronavirus in nasopharyngeal aspirate (NPA) samples ranges from 32% to 50% at the beginning of an illness and in stool samples is 97% at a mean of 14.2 days (*2*,*3*). The variation in sensitivity makes it difficult for the RT-PCR to be the standard criterion for diagnosis. Though the sensitivity is less than perfect, the assay can be used as a tool for early diagnosis. Until now, no data existed regarding the clinical course and outcome of SARS patients with NPA samples that were positive or negative for coronavirus by RT-PCR. We compared the epidemiologic, clinical, laboratory, and radiologic differences between RT-PCR–positive SARS and RT-PCR–negative SARS samples. We also looked for possible microbiologic evidence of coronavirus infection in RT-PCR–negative patients.

## Methods

### Patients

Two hundred sixty-seven patients fulfilling CDC case definition for suspected or probable SARS were admitted to the isolation wards of the Princess Margaret Hospital from February 26, 2003, to March 31, 2003. RT-PCR on NPA became available to us in mid-March. We included in our study 218 patients who had nasopharyngeal RT-PCR performed at illness onset.

### Investigation

Routine hematologic, biochemical, and microbiologic tests were performed for all patients. NPA samples were examined by rapid immunofluorescence antigen detection methods for viral cell culture and for common respiratory virus, including influenza viruses A and B; adenovirus; respiratory syncytial virus; and parainfluenza virus types 1, 2, and 3. Sputum samples were screened for bacterial and mycobacterial infection by conventional microscopic identification (Gram staining and acid-fast staining) and culture methods (blood, chocolate, MacConkey, and Löwenstein-Jensen media). Serologic testing for *Mycoplasma pneumoniae*, *Chlamydia pneumoniae,* and *C. psittaci* was performed. Urinary antigen detection tests were used to detect *Legionella pneumophila* and *Streptococcus pneumoniae* in some patients. Paired serum samples were taken 10–14 days apart to assess the serologic response to coronavirus by immunofluorescence assay (IFA). All chest radiographs were classified according to their laterality and extent of involvement.

### Qualitative RT-PCR Testing

The NPA sample collected from patients was added into a sterile vial containing 2 mL of viral transport medium and then transported on ice (4°C) to the Public Health Laboratory Centre, Government Virology Unit (GVU) of Hong Kong. Total RNA from 140 μL of each NPA sample was extracted by a QIAamp viral RNA Mini kit (QIAGEN, Valencia, CA), as instructed by the manufacturer and eluted in 60 μL of buffer. A total of 4.2 μL of eluted RNA was reverse-transcribed with use of reverse transcriptase (Applied Biosystem, Foster City, CA) in a 20-μL reaction containing 2.5 μM (final concentration) of random hexamer. The mixture was incubated at room temperature for 10 min and then at 42°C for 15 min. The reaction was stopped at 95°C for 5 min and then chilled in ice. The primers used for amplification, COR-1 and COR-2, were targeted at the coronavirus polymerase gene designed by GVU: sense 5′ CAC CGT TTC TAC AGG TTA GCT AAC GA 3′and antisense 5′ AAA TGT TTA CGC AGG TAA GCG TAA AA 3′, with expected product size of 311 bp. Five microliters of cDNA was amplified in 45 μL of master mixture containing 5 μL of 10X PCR buffer (Amersham Pharmacia Biotech, Piscataway, NJ), 1 μL of 2 5 mM extra MgCl_2_, 4 μL of deoxynucleoside triphosphates (dNTPs) (2.5 mM each), 0.5 μL of each primer, 0.3 μL of Taq polymerase (5 U/mL), and 33.7 μL of molecular grade water. One positive control and one negative control were included in each PCR assay. Reactions were performed in a thermocycler (GeneAmp PCR System 9700, Applied Biosystem) with the following conditions: at 94°C for 3 min, followed by 45 cycles of 94°C for 30 s, 60°C for 30 s, 72°C for 1 min, and 72°C for 7 min. PCR products were analyzed by gel electrophoresis.

### Treatment

All patients received treatment according to a standard protocol. Either a β-lactam plus β-lactamase inhibitor or third-generation cephalosporin in combination with a macrolide or a fluoroquinolone was given to the patient at admission. Per the recommendation of the Hospital Authority of Hong Kong, an antiviral drug (ribavirin 24 mg/kg/day intravenously, together with hydrocortisone 10 mg/kg/day) was administered if the symptoms did not respond within 48 h (The recommendations were found available at: URL: http://www.ha.org.hk/hk/hesd/nsapi/?Mlval=ha_view_content&c_id=122711&lang=E. However, the recommendations have since changed and are available at: URL: http://www.ha.org.hk/hesd/nsapi/?MIval=ha_view_content&c_id=123510&hesd_lang=E). Methylprednisolone, in the form of two to three pulsing doses of 500 mg to 1,000 mg a day intravenously, was administered to those with a persistent fever, radiologic evidence progression of lung infiltrates, or signs of respiratory distress despite the initial antiviral-hydrocortisone combination.

### Statistical Analysis

Bivariate analysis was performed for epidemiologic, clinical, laboratory, radiologic data, and outcomes by using RT-PCR results as the dependent variable. Data of continuous variables were expressed as mean and standard deviation. Chi-square test was used for categorical variables, and the unpaired Student t test was performed for continuous variables. All significant factors for death with a p value <0.1 were pooled into a multivariate logistic regression model with backward stepwise analysis to identify the independent predictors for the clinical outcome. A p value <0.05 (two-tailed) was assumed to be statistically significant. All analyses were performed with the SPSS version 10.0 software (SPSS Inc, Chicago, IL).

## Results

### Demographic Findings

On admission, nasopharyngeal RT-PCR was performed on 90 male and 128 female patients (mean age 39.6 ± 14 years). All patients, except six, were Chinese; two were Indonesian, and four were Filipino. Twenty-one of the patients (10%) were healthcare workers, including 5 clinicians, 9 nurses, 5 ward assistants, and 2 allied health workers who worked in the SARS wards. Forty-one patients (19%) reported having recently traveled to SARS-endemic areas in the 2 weeks before admission; the most common areas visited were in the southern part of China. Our cohort consisted of patients (46.8%) from a local housing estate, the Amoy Gardens. Ten patients (4.6%) had one or more coexisting medical problems: diabetes mellitus (3 cases), a history of cerebrovascular disease (4 cases), ischemic heart disease (3 cases), chronic rheumatic heart disease (1 case), hypertrophic obstructive cardiomyopathy (1 case), sick sinus syndrome (1 case), cirrhosis of the liver secondary to chronic hepatitis B (1 case), bronchiectasis (1 case), end-stage renal disease (1 case), Sjögren syndrome (1 case), and nasopharyngeal carcinoma (1 case). The proportions of patients from Amoy Gardens that were RT-PCR positive (48%) and negative (45.7%) were not significantly different (p = 0.76). Likewise, the proportions of healthcare workers (p = 0.28) or patients with coexisting conditions (p = 0.83) did not differ significantly.

### RT-PCR Results

NPA samples for RT-PCR were taken from all patients at admission; samples from 156 patients (71.6%) were positive. The mean time from disease onset to sample collection was 4.4 ± 2.3 days. No significant difference in the mean sampling time was found between RT-PCR–positive or –negative patients. The optimal time for sample collection was day 8–10 when 13 of 14 patients (92.9%) were positive (Figure).

**Figure:**
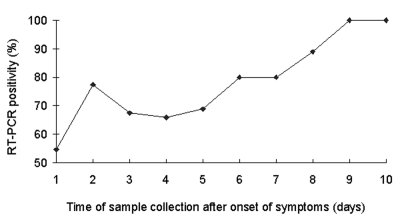
Percentage of reverse transcription polymerase chain reaction (RT-PCR) positivity at different times of sample collection after onset of symptoms.

### Symptoms and Laboratory Findings

The most common clinical features for both RT-PCR–positive and –negative cases included fever, chill, malaise, myalgia, cough, rigor, and headache (Table 1). Shortness of breath and dizziness were significantly higher in RT-PCR–positive patients in bivariate analysis. Vital signs taken on admission (temperature, heart rate, and systolic and diastolic blood pressure) were similar between the two groups. Common laboratory findings included anemia with hemoglobin level <12 g/dL (14.7%), lymphopenia with leukocyte count <4 x 10^9^/L (72%), thrombocytopenia with platelet count <150 x 10^9^/L (52.3%), hypokalemia with plasma potassium level <3.5 mmol/L (41.3%), hyponatremia with plasma sodium level <135 mmol/L (61.5%), and elevated levels of lactate dehydrogenase >230 U/L (46.6), alanine transaminase > 40 U/L (30.8%), and C-reactive protein (77.8%). By bivariate analysis, lymphopenia and elevated lactate dehydrogenase level on admission were significantly different between RT-PCR–positive and –negative patients (Table 2).

**Table 1:** Symptoms of 218 patients with severe acute respiratory syndrome^a^

Symptoms	Positive RT-PCR for coronavirus n=156 (%)	Negative RT-PCR for coronavirus n=62 (%)	p value
Time from symptoms onset to sample collection (days)	4.5 ± 2.2	4.2 ± 2.4	0.366
Fever	155 (99)	60 (97)	0.139
Chill	120 (77)	51 (82.3)	0.388
Malaise	103 (66)	34 (54.8)	0.123
Myalgia	84 (53.8)	30 (48.4)	0.467
Cough	66 (42.3)	27 (43.5)	0.867
Rigor	65 (41.7)	27 (43.5)	0.800
Headache	49 (31.4)	25 (40.3)	0.210
Anorexia	37 (23.7)	14 (22.6)	0.858
Sputum	28 (18)	13 (21)	0.607
Shortness of breath	36 (23)	5 (8.1)	**0.012**
Dizziness	23 (14.7)	17 (27.4)	**0.029**
Diarrhea	22 (14.1)	9 (14.5)	0.937
Sore throat	21 (13.5)	10 (16.1)	0.611
Runny nose	15 (9.6)	9 (14.5)	0.297
Chest pain	13 (8.3)	8 (12.9)	0.302
Vomiting	12 (7.7)	5 (8)	0.926
Palpitation	2 (1.3)	2 (3.2)	0.320
Hemoptysis	1 (0.6)	1 (1.6)	0.497
Confusion	1 (0.6)	1 (1.6)	0.497

^a^Fisher exact test was applied if p value was <5; RT-PCR, reverse transcription polymerase chain reaction; p values in bold are significant.

**Table 2:** Vital signs and laboratory findings in 218 patients with severe acute respiratory syndrome

	Positive RT-PCR for coronavirus (n = 156)	Negative RT-PCR for coronavirus (n = 62)	p value
Vital sign upon admission			
Temperature (°C)	38.5 ± 0.9	38.4 ± 0.9	0.774
Heart rate	95 ± 14	98 ± 16	0.571
Systolic blood pressure	127 ± 18	130 ± 19	0.503
Diastolic blood pressure	71 ± 11	73 ± 12	0.450
Laboratory findings upon admission			
Hemoglobin level (g/dL)	13.3 ± 1.4	13.0 ± 1.6	0.160
Leukocyte count (x 10^9^/L)	5.5 ± 2.7	5.5 ± 1.9	0.954
Neutrophil count (x 10^9^/L)	4.3 ± 2.6	4.2 ± 2.3	0.885
Lymphocyte count (x 10^9^/L)	0.8 ± 0.3	0.9 ± 0.3	*0.045*
Platelet count (x 10^9^/L)	155 ± 55	166 ± 50	0.137
Prothrombin time (sec)	12 ± 2	12 ± 1	0.396
Activated partial thromboplastin time (sec)	35 ± 10	33 ± 5	0.094
Sodium level (mmol/L)	134 ± 4	134 ± 3	0.423
Potassium level (mmol/L)	3.6 ± 0.5	3.5 ± 0.4	0.787
Urea level (mmol/L)	3.7 ± 1.8	3.6 ± 4	0.200^a^
Creatinine level (mmol/L)	74 ± 19	80 ± 79	0.885^a^
Albumin level (g/L)	37± 4	38 ± 5	0.112
Globulin (g/L)	33 ± 5	33 ± 4	0.737
Bilirubin (mmol/L)	9 ± 6	8 ± 5	0.798
Alkaline phosphatase (IU/L)	75 ± 58	67 ± 33	0.245^a^
Alanine aminotransferase (IU/L)	43 ± 41	33 ± 30	0.051^a^
Creatinine phosphokinase (IU/L)	422 ± 1987	189 ± 391	0.118^a^
Lactate dehydrogenase (IU/L)	287 ± 141	208 ± 67	**0.001^a^**

^a^Comparison made after log-transformation of data; RT-PCR, reverse transcription polymerase chain reaction; p values in bold are significant.

### Serologic Test Results

Eighty-seven NPA RT-PCR–positive patients and 33 RT-PCR–negative patients had serologic tests on their paired serum samples 10–14 days apart. Of the positive RT-PCR patients, 74 patients (85.1%) had total antibodies detected by IFA, while serologic tests for 25 patients (75.8%) in the RT-PCR–negative group were positive. Results for 13 patients in the RT-PCR–positive group and 8 patients in the RT-PCR–negative group were negative.

### Radiologic Findings

Initial chest radiographs for 210 patients (96.3%) were abnormal. Sixty-five (41.7%) RT-PCR–positive patients and 13 (21%) RT-PCR–negative patients had bilateral chest involvement shown by radiograph. Multifocal radiologic involvement was found in 74 (47.4%) RT-PCR–positive patients and 15 (24.2%) RT-PCR–negative patients. By bivariate analysis, RT-PCR–negative patients were less likely to have abnormal bilateral (p = 0.01) and multifocal (p = 0.003) radiographs.

### Outcomes

The overall 30-day mortality rate was 10.1% (22 patients). Fifty-two (23.9%) patients required intensive-care unit (ICU) admission, and 43 patients (19.7%) needed mechanical ventilation. In nine (4.1%) patients, acute renal failure further complicated SARS. When compared to the RT-PCR–negative patients, the RT-PCR–positive patients were more likely to need treatment in the ICU (p = 0.002), require mechanical ventilation (p = 0.008), and die (p = 0.044) (Table 3).

**Table 3:** Clinical progress and outcome on day 30 after admission

Clinical progress/outcome	Positive RT-PCR for coronavirus n=156 (%)	Negative RT-PCR for coronavirus n=62 (%)	p value	
Patients requiring ICU care	46 (29.5)	6 (9.7)	0.002	
Patients requiring mechanical ventilation	38 (24.4)	5 (8.1)	0.008^a^	
Patients developing acute renal failure	8 (5.1)	1 (1.6)	0.451^a^	
Death	20 (12.8)	2 (3.2)	0.044^a^	

^a^Fisher exact test was applied if number was <5; RT-PCR, reverse transcription polymerase chain reaction.

### Predictors of Mortality

Admission parameters, including epidemiologic data, vital signs, and laboratory and chest radiographic findings, were analyzed separately. By bivariate analysis, factors associated with death were age >60 (p = 0.037), male sex (p = 0.007), major coexisting medical conditions (p = 0.001), shortness of breath (p = 0.005), total leukocyte count >4.0 x 10^9^/L (p = 0.041), bilateral chest radiographic involvement (p = 0.046), RT-PCR positivity on NPA samples (p = 0.034), and pulsing doses of steroid (p = 0.001). By multivariate analysis, independent predictors of 30-day mortality were RT-PCR positivity on NPA samples (odds ratio [OR] 6.4; 95% confidence interval [CI] 1.1 to 38.0; p = 0.038), shortness of breath on admission (OR 3.9; 95% CI 1.2 to 12.3; p = 0.02), presence of important coexisting condition (OR 13.4; 95% CI 3.1 to 58.2; p = 0.001), total leukocyte count >4.0 x 10^9^/L (OR, 6.94; 95% CI 1.18 to 41.6; p = 0.033), and pulsing doses of methylprednisolone (OR 26.0; 95% CI 4.4 to 154.8; p = 0.001) (Table 4).

**Table 4:** Multivariate analysis on risk factors associated with 30-day mortality^a^

Risk factors	Adjusted OR (95% CI)	p value
Significant coexisting conditions	13.4 (3.1 to 58.2)	0.001
Shortness of breath on admission	3.9 (1.2 to 12.3)	0.020
Total leukocyte count >4.0 x10^9^/L at admission	6.94 (1.18 to 41.6)	0.033
Positive RT-PCR on NPA	6.4 (1.1 to 38.0)	0.038
Use of pulsing doses of steroid	26.0 (4.4 to 154.8)	0.001

^a^OR, odds ratio; CI, confidence interval; RT-PCR, reverse transcription polymerase chain reaction; NPA, nasopharyngeal aspirates.

## Discussion

In general, the epidemiologic background, clinical presentation, laboratory findings, and radiologic findings of our patients were similar to those of other reports (*3*–*5*). The clinical features of our cohort were rather nonspecific, with fever, chills, malaise, and myalgia being the most common. Radiologic features of our patients were similarly nonspecific. Anemia, lymphopenia, and thrombocytopenia were common on admission. These symptoms might reflect peripheral consumption or bone marrow suppression by the infection. Elevation of alanine transaminase, C-reactive protein, and lactate dehydrogenase levels was frequently observed; this finding might indicate extensive tissue damage.

Currently, the definition of SARS is mainly clinical, and diagnosis is made by exclusion of pneumonia from other known causal agents. However, patients with similar clinical scenarios may not be infected by the same agent, and placing them in the same location may spread infection. Unfortunately isolating every patient is not possible, especially with a large cohort. An early, rapid, and reliable test is needed. After coronavirus was recognized as the putative agent for SARS, diagnostic tests have burgeoned rapidly over the past 2 months. However, serologic tests cannot offer an early diagnosis since, despite their remarkable specificity, they require approximately 3 weeks for the total antibodies to become detectable (*2*). Electron microscopy and viral culture are not sensitive and convenient enough for general use. Inevitably, clinical characteristics are used solely for the diagnosis of SARS, despite the condition’s nonspecific nature.

RT-PCR for coronavirus on NPA samples appears to be the best supportive test for an early and firm diagnosis. However, the sensitivity of this test varies, and standardization of the test has not been unified. The test we used was qualitative and had good sensitivity (71.6%). In our study, the mean time between onset of symptoms and sample collection was 4.3 days. Peiris et al. reported that the sensitivity for RT-PCR was 32% at a mean 3.2 days after onset (*3*). In our study, the best time for sampling was on days 8 to 10 after onset of symptom. During that period, >90% of the samples were positive. However, since the condition of SARS patients deteriorated both clinically and radiologically during this time, waiting 8–10 days to make a firm diagnosis or to plan for appropriate therapy is not possible (*3*). The mean time of sample collection did not differ significantly between RT-PCR–positive or –negative patients, suggesting that the difference in outcomes between these two groups could not be explained by the discrepancy in the duration of their symptoms before admission. Whether the difference in primers used in the RT-PCR testing or infection by a another agent, such as metapneumovirus or *Chlamydia* species (*6*), could also contribute to the discrepancy in sensitivity is not known.

Peiris et al. reported that the sensitivity of stool samples for RT-PCR tests was 97% (mean of 14.2 days) (*3*). In our study, the overall positive rate was 30%. However, RT- PCR was performed on stool specimens only when diarrhea developed in patients. Inconsistency in the timing of sample collection might also contribute to the low positive rate. In patients with negative RT-PCR results on NPA samples, none had a stool sample positive by RT-PCR. Hence, RT-PCR on stool specimen could not provide an early diagnosis of coronavirus infection in our cohort.

Patients with a positive RT-PCR result on admission had adverse outcomes in term of survival, ICU care, and assisted ventilation, when compared to patients with negative RT-PCR results. Therefore, the patients with overwhelming disease had more viral shedding, which may have been more readily detected. Despite the satisfactory sensitivity that we demonstrated, the test might not provide the information for quantitative analysis. Hence, a negative result might not represent low viral load in patients and vice versa. A quantitative RT-PCR could give us some idea as to the correlation between the viral concentration and disease progression. Peiris et al. reported that the maximal viral replication by quantitative RT-PCR occurred by approximately day 10, but the clinical worsening seemed to lag behind this peak (*3*). Although we could not quantify the maximal viral shedding, the maximal RT-PCR positivity did fall on approximately day 10. Peiris et al. also demonstrated that an initial positive RT-PCR result had no correlation to development of an acute respiratory distress syndrome. In our multivariate analysis model, however, initial RT-PCR positivity on NPA was an independent predictor for a worse outcome, rather than a previously reported factor, such as a coexisting condition (*3*–*5*). Although quantitative RT-PCR was not performed on samples from our patients, since the test was not available at that time, a qualitative RT-PCR result might alert the clinician to watch out for possible clinical deterioration, especially when the former test was in its infancy for common use. The relationship between viral load on NPA and outcome should be further investigated.

The clinical outcomes of RT-PCR–positive patients are worse in general when compared to RT-PCR–negative patients, and their chest radiographs show more bilateral and multifocal haziness. A higher level of lactate dehydrogenase was observed in the RT-PCR–positive patients, which might indicate more extensive lung tissue injury, as indicated in other SARS patients with poor outcome (*4*). Whether the lower lymphocyte count in RT-PCR–positive patients suggests more extensive viral infection remains to be clarified. Use of pulsing doses of methylprednisolone could result in clinical improvement and the resolution of radiologic infiltration in some of our patients. However, its immunosuppressive effect could also predispose a patient to secondary nosocomial infection and subsequent death.

How to handle negative results in RT-PCR testing is a problem. In accordance with the World Health Organization’s (WHO) recommendations, a negative result has at least two possibilities (*7*). First, it may indicate a false-negative result caused by low viral load or inappropriate timing of sample collection. Second, another infectious or a noninfectious agent may be the cause of SARS instead of coronavirus. Finally, a negative RT-PCR result on admission may indicate early elimination of the virus by an effective and harmonious immunologic response. Serologic tests are thus important in identifying SARS infections, although the diagnosis could not be made early enough to prompt an appropriate action. In our patients, RT-PCR and serologic results were in concordance. The sensitivity of RT-PCR test was 74.7% when an antibody test was used as a standard, which can be explained by the variation in the technique and timing of sampling. The optimal timing for the RT-PCR test is unknown. The problem of finding an appropriate sampling time was taken in account for the RT-PCR–positive patients with negative serologic results, since they suggested that antibodies could be detected at 21 days (*8*) instead of 10–14 days, as in our cohort. In addition, the PCR test may be overly sensitive, which may be why WHO has advised clinicians to confirm a positive RT-PCR result by repeating the test with the original sample or testing the sample in a secondary laboratory so as to increase its specificity (*8*).

Results of both RT-PCR and antibody tests were negative in eight patients; all of these patients had signs and symptoms that were clinically, radiologically, or biochemically well-matched with SARS, and they were given treatment, including ribavirin and steroid. Pulsing doses of steroid was also used in two of these patients. In addition to the sample timing, these patients could represent a milder spectrum of the disease with little antibody stimulation or inconspicuous coronavirus RNA level, or simply infection other than coronavirus. Antibody production may have been suppressed because of steroid administration.

Because RT-PCR testing has not been standardized, the test still varies in sensitivity and specificity, and we are still confronted with a clinical dilemma in terms of infection control and management. Furthermore, the controversy of medication in the management of SARS has never been settled. Current treatment guidelines proposed by the Hospital Authority of Hong Kong are still in use despite the adverse effects of the suggested treatment (The recommendations were found available at: URL: http://www.ha.org.hk/hk/hesd/nsapi/?Mlval=ha_view_content&c_id=122711&lang=E.)Without a reliable and rapid RT-PCR test for diagnosis, patients mislabeled as having SARS will be offered treatment that they do not need.

Our results indicate that a positive nasopharyngeal RT-PCR result on admission, from the current standard, should raise the possibility of SARS in appropriate clinical settings and should alert the clinician of the possible clinical deterioration of the patient. Furthermore, clinicians should consider repeating the qualitative RT-PCR test or performing quantitative RT-PCR test for a previously RT-PCR–negative patient. Drug treatment for this group of patients may be withheld or delayed, especially if effective and reliable treatment has not been found.
